# Antimicrobial-Resistant Bacteria and Prescription of Antibiotics at a Tertiary Care Hospital in Riyadh, Saudi Arabia

**DOI:** 10.7759/cureus.12098

**Published:** 2020-12-15

**Authors:** Anbar Aldawsari, Kamilia Tawfik, Ibrahim Al-Zaagi

**Affiliations:** 1 Pharmaceutical Services, King Saud Medical City, Riyadh, SAU; 2 Pharmacy, Riyadh Elm University, Riyadh, SAU

**Keywords:** antibiotic, infections, infection, multidrug resistance organism, prescription, tertiary hospital

## Abstract

Purpose

The purpose of the study was to assess the bacterial resistance and annual antibiotic consumption at a tertiary care hospital in Riyadh, Saudi Arabia over a two-year period.

Methods

This retrospective cohort study was conducted at a tertiary care hospital in Riyadh, Saudi Arabia from January 1, 2016, to December 31, 2017.

Results

The results showed that there was no significant difference between 2016 and 2017 data regarding patient characteristics like bed occupancy rate, the average length of stay, and the number of admissions; the same was true for bacterial characteristics like the number of bacteria, percentage of isolates in the group, and multidrug resistance (MDR) percentage (p: >0.05). Between 2016 and 2017, there was a slight reduction in the sensitivity of *Escherichia*​​​* coli* (*E. coli*) carbapenem-resistant Enterobacteriaceae (CRE) (97%, 86%) and *Klebsiella pneumoniae *(*K. pneumoniae*) CRE (80%, 76%) towards colistin. There was also a decrease in the sensitivity of *Acinetobacter baumannii* (*A. baumannii*) multidrug-resistant organism (MDRO) from 42% to 29% against tigecycline, but an increase in the sensitivity of *K. pneumoniae *CRE (33%, 50%) and *E. coli *CRE (76%, 82%). The percentage of MDR strains in gram-positive bacteria showed that more than half of* Staphylococcus aureus *(*S. aureus*) were methicillin-resistant (61%, 59%) in 2016 and 2017 respectively. There was a reduction in the percentage of MDR strains in some gram-negative bacteria like *Pseudomonas aeruginosa* (*P. aeruginosa*) MDRO (24%, 19%),*E. coli* extended-spectrum beta-lactamases (ESBL) (56%, 50%), *E. coli* CRE (4%, 1%), *K. pneumoniae *CRE (49%, 33%), *A. baumannii *CRE (90%, 76%), and *Proteus mirabilis​​​​*​​​ (*P. mirabilis)* ESBL (54%, 50%).

Conclusion

MDRO bacteria are very common in the hospital where the study was conducted. Immediate action is required to tackle this problem.

## Introduction

The first antibiotic, penicillin, was discovered by Alexander Fleming in 1928 [1]. Since then, doctors have been able to save millions of lives. However, it did not take long for the threat of infections to return when some bacteria strains were found not susceptible to penicillin. Before penicillin was started to be used clinically to treat infections in 1943, bacterial resistance had already been described by some researchers in 1940. In 1962, methicillin-resistant *Staphylococcus aureus* (MRSA) was isolated in the United States (US). But, on the other hand, new antibiotics also were being discovered, which made people feel safe from threats of infection [2]. Recently, the development of resistance to antibiotics has become faster, and the emergence of strains for which there is no treatment, such as carbapenem-resistant Enterobacteriaceae (CRE) and colistin-resistant bacteria, have been reported from various countries [3]. To handle this issue, there have been initiatives to develop new antibiotics, and an emphasis has been placed on the rational use of existing ones. The aim of this study was to assess bacterial resistance and annual antibiotic consumption at a tertiary hospital in Riyadh, Saudi Arabia over a two-year period.

## Materials and methods

This was a retrospective cohort study conducted to investigate the prevalence of antibiotic-resistant bacteria over a period of two years and the prescription of antibiotics during this period at a tertiary hospital. The study involved data collected from King Saud Medical City, a tertiary care hospital in the city of Riyadh, Saudi Arabia. The hospital has a capacity of 1,400 beds, and the service covers most of the medical specialties. The data included in this study were recorded for two years from January 2016 to December 2017. All bacterial susceptibility testing were sent to the laboratory department of the concerned hospital.

Data collection procedure

Bacteria Isolate Results

The data of the bacterial cultures, which included the bacteria isolates and their sensitivity to antibiotics, were collected from the hospital’s laboratory department. The data were obtained from the health information system or the bacteriology section reports and antibiograms.

Antibiotics Prescription/Consumption

Data concerning the antibiotics consumed in the hospital were also collected from the hospital information system (Medisys®) for the concerned period. 

Hospital Infection Control Measures

Relevant data were also collected from the infection control department. The included data described reports containing data about some activities conducted to minimize the chance of infection spread.

Statistical analysis

Data were analyzed using the SPSS Statistics v22 (IBM, Armonk, NY). Descriptive statistics and the Mann-Whitney test for the comparison between the variables were used. P-values of less than 0.05 were considered statistically significant.

## Results

The results showed that there was no significant difference between the 2016 and 2017 data regarding patient characteristics like bed occupancy rate, the average length of stay, and the number of admissions; the same was true for bacterial characteristics like the number of bacteria, percentage of isolates in the group, and multidrug resistance (MDR) percentage (p: >0.05) (Table 1).

**Table 1:** Distribution of values in the studied years regarding bacteria and patient characteristics M: median; IQR: interquartile range; MDR: multidrug resistance

	2016	2017	Mann-Whitney	P-value
Bacteria				
Number of bacteria	374.57 ± 301.96	407.14 ± 366.37	0.18	0.854
	M = 330.50	M = 473.0		
	IQR = 95.75-530.75	IQR = 99.75-766.25		
% in group	46.71 ± 28.25	43.21 ± 26.73	0.34	0.73
	M = 47.50	M = 42.5		
	IQR = 23.50-62.0	IQR = 20.75-58.25		
MDR %	8.42 ± 6.80	7.23 ± 7.48	0.68	0.493
	M = 7.40	M = 9.60		
	IQR = 2.12-11.95	IQR = 2.02-15.55		
Patients				
Bed occupancy rate	75.79 ± 3.85	71.1 ± 9.38	0.83	0.402
	M = 75.70	M = 75.60		
	IQR = 73.15-78.95	IQR = 68.67-76.67		
Average length of stay	7.85 ± 0.52	8.25 ± 1.24	0.81	0.418
	M = 7.95	M = 8.35		
	IQR = 7.52-8.10	IQR = 7.22-9.22		
Number of admissions	3602.58 ± 299.89	3749.66 ± 556.53	1.73	0.083
	M = 3599.50	M = 3914		
	IQR = 3329-3824.25	IQR = 3548.75-4070.25		

**Figure 1:**
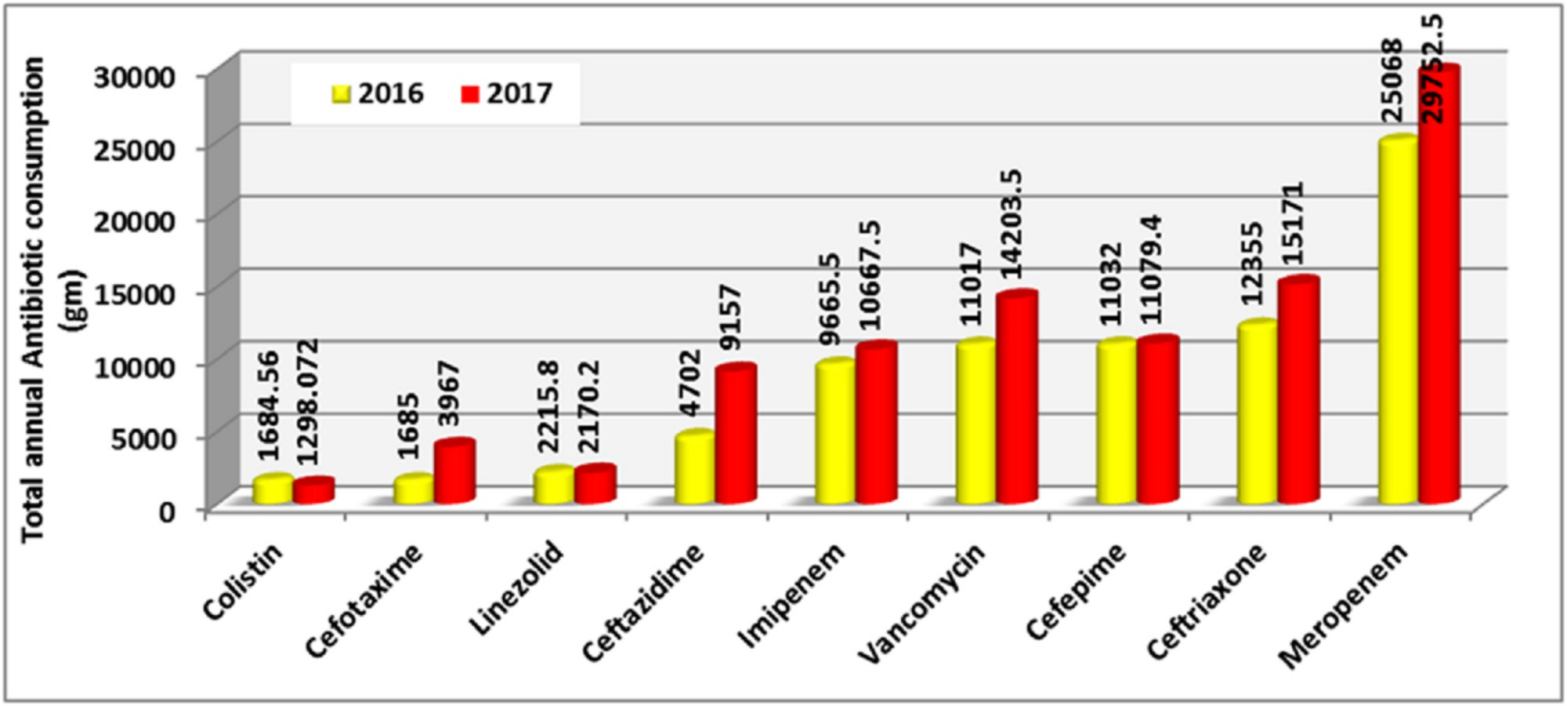
Total annual consumption (gm) of antibiotics in 2016 and 2017 - chart 1

**Figure 2:**
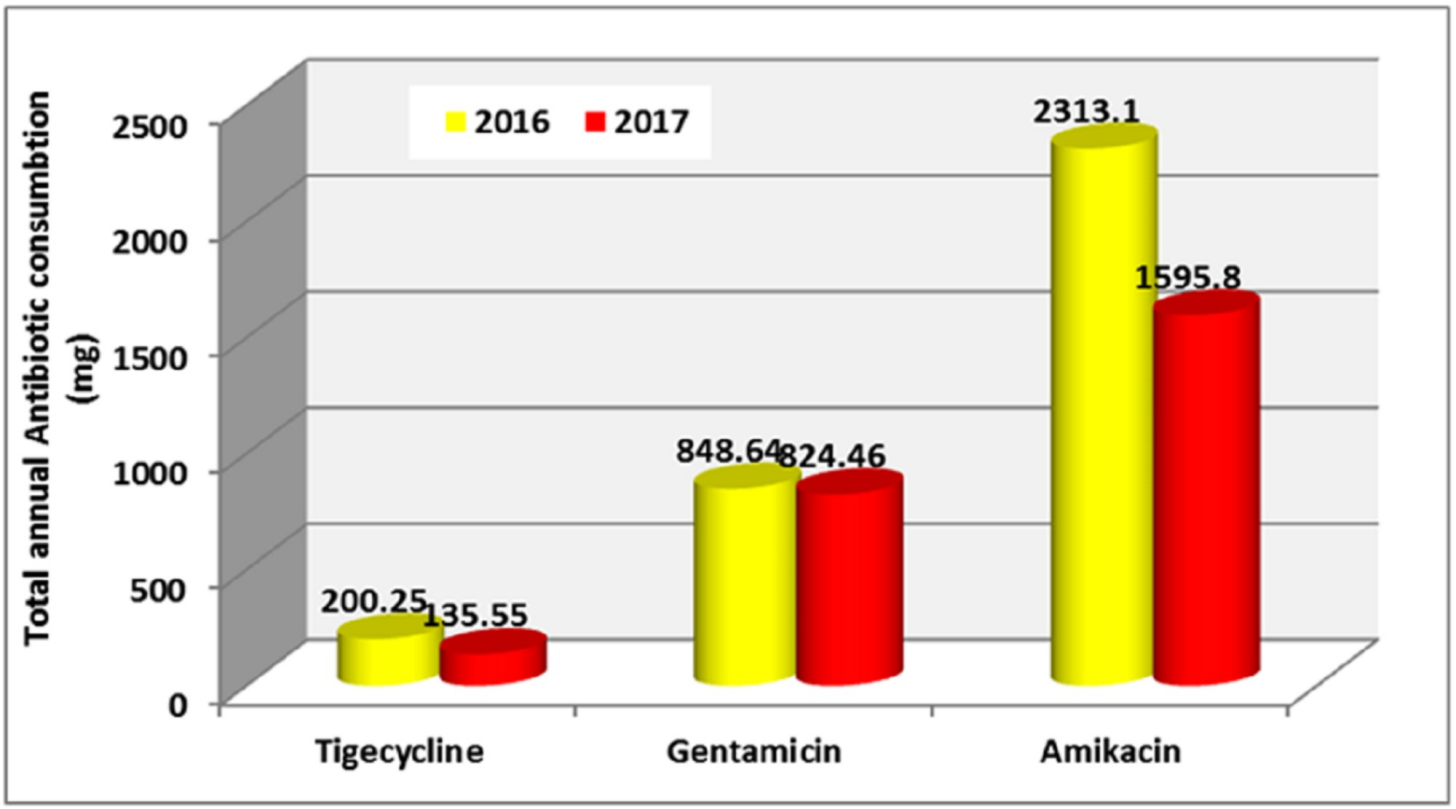
Total annual consumption (mg) of antibiotics in 2016 and 2017 - chart 2

An overall increase in the usage of antibiotics was detected. There was a consistent increase in the use of carbapenems and cephalosporins (third-gen.). The exception to this was the colistin, tigecycline, amikacin, and linezolid where there was a slight decrease. The amount of cefepime and gentamicin remained almost unchanged. Other agents showed fluctuation in their usage (Figure 1, Figure 2).

**Figure 3:**
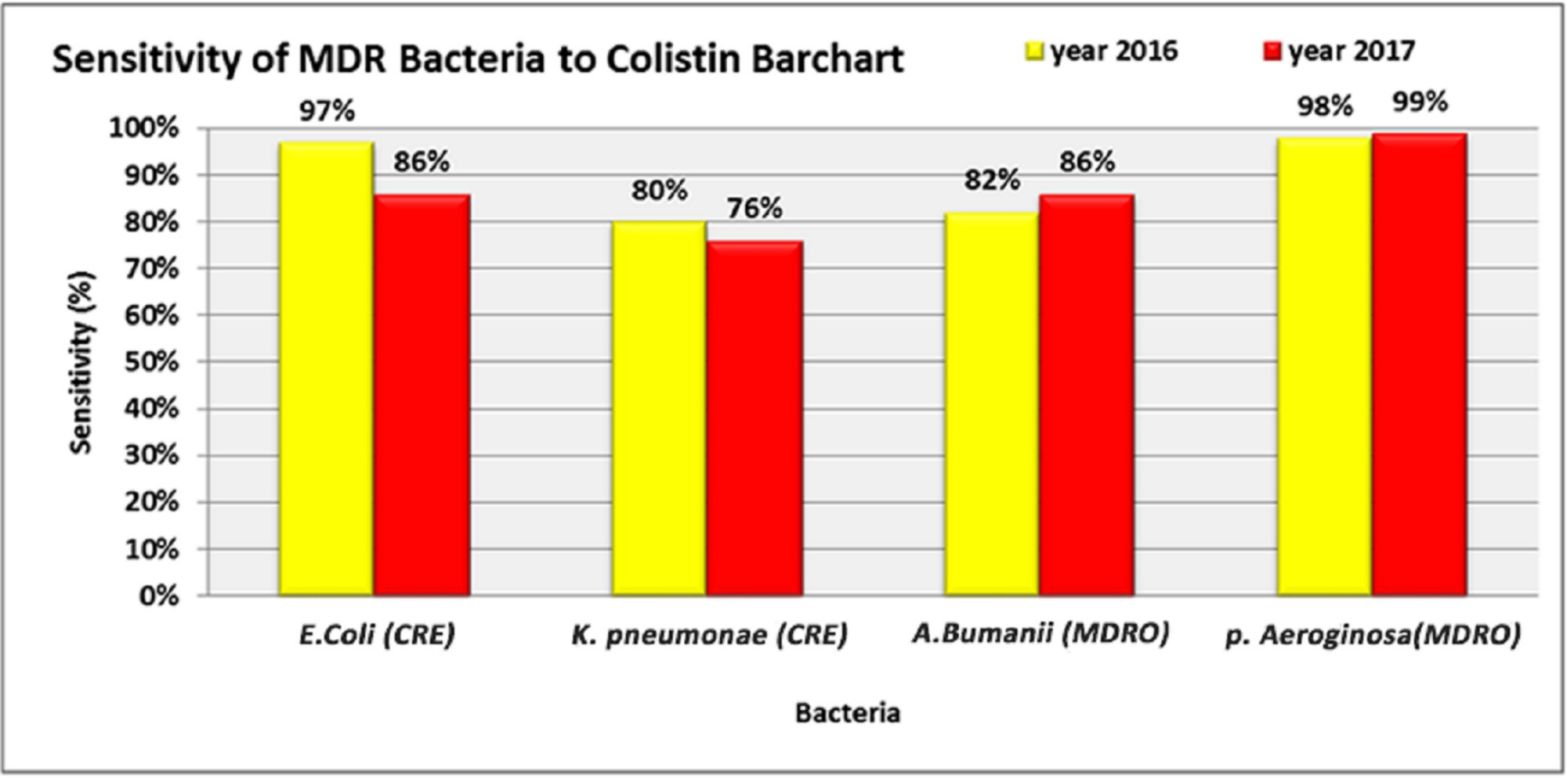
Percentage of MDR gram-negative bacteria against colistin CRE: carbapenem-resistant Enterobacteriaceae; MDRO: multidrug-resistant organism

Data collection revealed a faulty method of calculation of the prescribed antibiotics, which made it difficult to quantify or compare. The WHO metrics for quantifying the defined daily dose (DDD) were meant to be used in this study. However, because the query available in the hospital information system did not provide the data on how many days each antibiotic was used for, it was not possible to calculate the results for those metrics. Regarding colistin, between 2016 and 2017, there was a slight reduction in the sensitivity of *Escherichia coli* (*E. coli*) CRE (97%, 86%) and *Klebsiella pneumoniae* (*K. pneumoniae*) CRE (80%, 76%). In contrast, there was a slight increase in the sensitivity of *Acinetobacter baumannii* (*A. baumannii*) multidrug-resistant organism (MDRO) (82%, 86%) and *Pseudomonas aeruginosa* (*P. aeruginosa*) (98%, 99%) (Figure 3).

**Figure 4:**
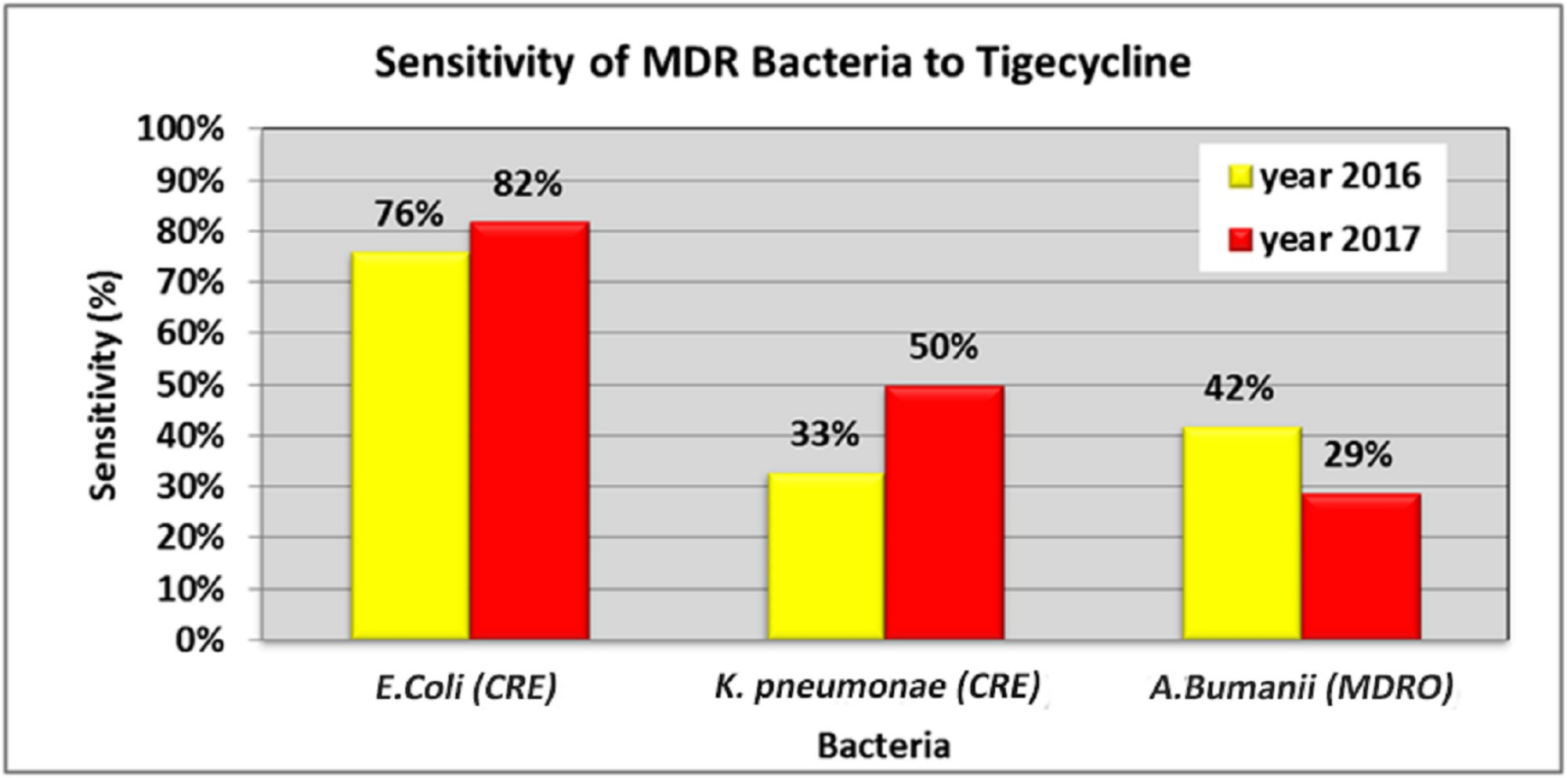
Percentage of MDR gram-negative bacteria against tigecycline CRE: carbapenem-resistant Enterobacteriaceae; MDRO: multidrug-resistant organism

For tigecycline, there was a decrease in the sensitivity of *A. baumannii* MDRO from 42% to 29%. However, there was an increase in the sensitivity of *K. pneumoniae* CRE (33%, 50%) and *E. coli* CRE (76%, 82%) from 2016 to 2017 (Figure 4).

**Figure 5:**
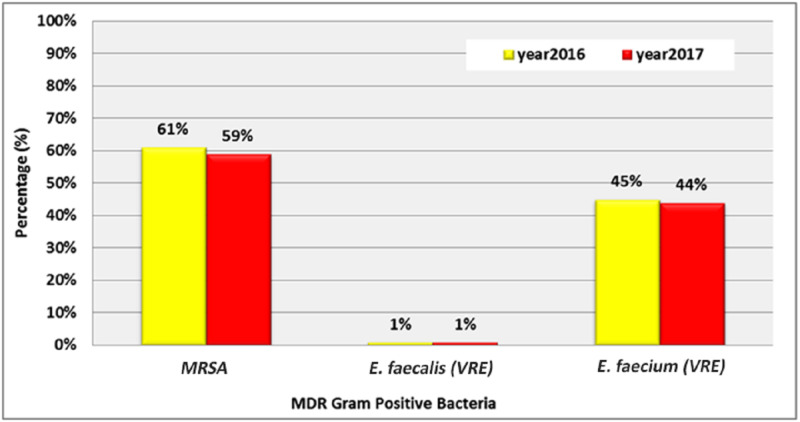
Distribution of values in the studied years regarding the percentage of MDR gram-positive bacteria MRSA: methicillin-resistant Staphylococcus aureus; VRE: vancomycin-resistant enterococci

The percentage of MDR strains in gram-positive bacteria showed that more than half of *Staphylococcus aureus *(*S. aureus*) are gram methicillin-resistant (61%, 59%). Only 1% of *Enterococcus faecalis* (*E. faecalis*) bacteria were vancomycin-resistant enterococci (VRE) in both years. The percentage of VRE among *Enterococcus faecium* (*E. faecium*) in 2016 and 2017 were 45% and 44% respectively (Figure 5).

**Figure 6:**
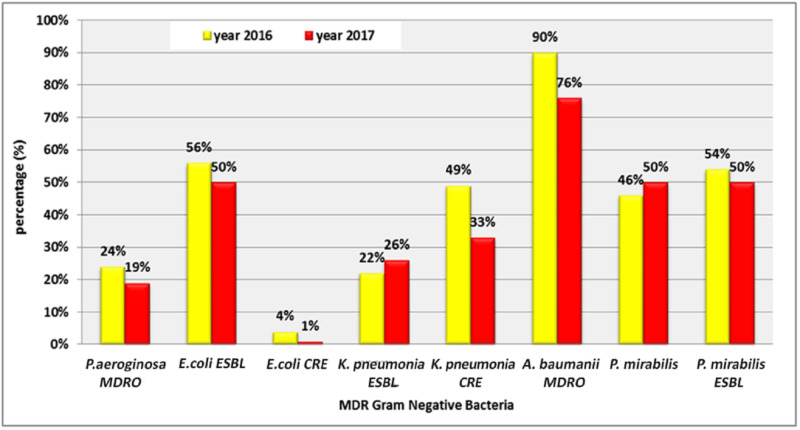
Distribution of the values in the studied years regarding the percentage of MDR gram-negative bacteria MDRO: multidrug-resistant organism; ESBL: extended-spectrum beta-lactamases; CRE: carbapenem-resistant Enterobacteriaceae

There was a reduction in the percentage of MDR strains in some gram-negative bacteria types such as *Pseudomonas aeruginosa* (*P. aeruginosa)* MDRO (24%, 19%), *E. coli *extended-spectrum beta-lactamases (ESBL) (56%, 50%), *E. coli* CRE (4%, 1%), *K. pneumoniae* CRE (49%, 33%), *A. baumannii* CRE (90%, 76%), and *Proteus mirabilis (P. mirabilis)* ESBL (54%, 50%). On the other hand, there was an increase in the percentage of MDR strains in *K. pneumonia*e ESBL (22%, 26%) (Figure 6).

## Discussion

WHO’s Global Antimicrobial Surveillance System (GLASS) has collected data relating to antibiotic resistance among half a million people with suspected bacterial infections across 22 countries. The most commonly documented resistant bacteria, in descending order, were *E. coli,* *K. pneumoniae*, *S. aureus*, *S. pneumonia,* and *Salmonella* species. Worldwide, among patients with suspected bloodstream infections, bacteria resistant to at least one of the most commonly administered antibiotics ranged from 0% to 82%, which represents an increasing economic burden. In the US alone, antibiotic-resistant pathogen-associated hospital-acquired infections (HAIs) cause nearly 100,000 deaths annually [4,5]. In our study, the insignificant difference between the two years in terms of the number, type of bacteria, and the corresponding tested antibiotics may be due to the increased awareness about antibiotic use and prescribing. Another integral reason is the strict measures in place at the hospital to control infections and cross-contamination. On the other hand, the results of the study showed that there were 12 antimicrobial-resistant (AMR) bacteria in 2016 and 12 AMR bacteria in 2017 as well. The majority of these isolates were gram-negative bacteria, which is consistent with the findings of another study carried out at a tertiary care facility in Riyadh where the gram-negative organisms were found to be the most common (82.2%) [6]. In agreement with these results, it was reported that high rates of resistance to commonly used antibiotics were from gram-negative bacteria [7]. These findings are also in line with the findings from another study, which reported that most of the causative pathogens for nosocomial infections were gram-negative bacteria and that they were resistant to various classes of antimicrobials [8].

Colistin is considered to be the last choice for eradicating gram-negative bacteria, and, therefore, it is reserved for those strains that are resistant to all other antibiotics. Several carbapenem-resistant bacteria such as *E. coli* CRE, *K. pneumoniae* CRE,* A. baumannii* MDRO, and *P. aeruginosa* MDRO were sensitive to colistin (97%, 80%, 82%, and 98% respectively). Apart from the change in *K. pneumoniae* CRE sensitivity (76%), which is considered as intermediate sensitive, a minor change in the sensitivity of other bacteria such as *E. coli* (CRE), *A. baumannii* (MDRO), and *P. aeruginosa* (MDRO) to colistin (86%, 86%, and 99%) was seen in 2017. These findings are similar to some studies that showed the spread of colistin resistance worldwide with varying levels. *A. baumannii *resistance to colistin has been reported from different regions such as Saudi Arabia (4.7%), Asia, Europe, North America, and South America (7%). Bulgaria and Spain showed even higher rates (16.7% and 19.1% respectively). Figures from Spain and Korea were even higher (40.7% and 30.6% respectively) [9]. The reduction in the usage of colistin and linezolid can be considered as a good practice to prevent or delay the resistance against these two antibiotics. This reduction was accompanied by the increase in the consumption of other groups of antibiotics such as cephalosporins and carbapenems. The reason behind saving these items is that they are considered the last choice for gram-negative and gram-positive bacterial infections [10]. In 2016, the MDR bacteria found were *E.coli* ESBL, *K. pneumoniae* ESBL, and *P. mirabilis*. The extensively drug-resistant (XDR) organisms found were MRSA, *E. faecalis* VRE, *E. faecium* VRE, *P. aeruginosa* MDRO, *E. coli* CRE, *K. pneumoniae* CRE, *A. baumannii* MDRO, and *P. mirabilis* ESBL In 2017, the MDRO bacteria found included *E. coli* CRE, *K. pneumoniae* CRE, *A. baumannii* MDRO, *P. mirabilis* ESBL, *Providencia stuartii* (*P. stuartii)*, and *Morganella morganii* (*M. morganii)*. The only two MDR found were *E. faecalis* and *K. pneumoniae* ESBL. In a tertiary facility in Riyadh, it was found that “*A. baumannii* isolates detected during the study period were almost resistant to all the drugs being tested. All the *A. baumannii* isolates would have been classified as XDR, i.e., resistant to three classes (all penicillin, cephalosporins, fluoroquinolones, and aminoglycosides) and carbapenems” [6].

According to the Code of Conduct of Healthcare Practitioners in Saudi Arabia published in December 2005, a pharmacist is not allowed to dispense any medication without a prescription written by a locally registered physician. However, this regulation was not strictly implemented and it was possible to obtain antibiotics even without a prescription. There were calls from healthcare professionals to rectify this issue [11]. Therefore, in 2018, the Ministry of Health reemphasized the regulations that prevent the dispensing of antibiotics without a prescription and introduced a new penalty for violating these regulations. In three countries, Saudi Arabia, Bahrain, and the United Arab Emirates (UAE), between 2012-2015, a report found the presence of the mcr-1 gene in four *E. coli* [12]. The Gulf Cooperation Council region harbors other rare and novel antibiotic-resistant mechanisms as well, for example, the appearance of *P. aeruginosa *ESBL and drug-resistant *K. pneumoniae* in Qatar and UAE respectively [13]. National surveillance carried out in Saudi Arabia has reported resistance to methicillin in 32% of *S. aureus, ​​*while there was a reported resistance to penicillin G (33%) and erythromycin (26%) in *S. pneumonia* [14]. Another study that showed MRSA in 18% of health workers demonstrated that the total prevalence of MRSA among health workers was 18% [15].

In Saudi Arabia, the factors behind the emergence of resistance can be summarized in the following points: a) non-optimized use of antibiotics, b) availability of over-the-counter antibiotics without prescription in Saudi community pharmacies, c) non-adherence to infection control measures, and d) the eagerness to satisfy patients. In 2010, the overuse of antimicrobial agents from four adult ICUs in Riyadh was reported, where meropenem was reported to be the most common one, followed by piperacillin-tazobactam. On the other hand, in the US, the highest use has been reported for carbapenems followed by antipseudomonal penicillins in 37 ICUs. Antibiotics without a prescription have been found to be dispensed in about 77.6% of the pharmacies in Riyadh [16]. In 2016, a study was by Leangapichart et al. regarding bacterial infections during the Hajj pilgrimage showed that travelers who returned from Hajj acquired ceftazidime resistance in more than 20% of *E. coli*, 30% of *K. pneumoniae*, and 50% of *P. aeruginosa* [17]. Regarding the hand hygiene compliance rate, in a study carried out at a hospital in Makkah in 2011, it was found to be about 50%. In Riyadh, controlling a nosocomial outbreak caused by carbapenem-resistant *K. pneumoniae* was significantly helped by effective hand hygiene compliance [16].

During the World Health Assembly in 2015, a Global Action Plan on AMR launched by WHO was ratified by most of the member states, including Saudi Arabia, and it consisted of five main pillars [18]: awareness improvement, knowledge strengthening, infection reduction, antimicrobial agents use optimization, and sustainable investment to support the need to find new drugs, investigating and diagnostic techniques, and vaccines. Patients’ attitudes to antibiotics and the average time taken by doctors for consultations are the most important criteria to measure how physicians try to satisfy their patients. It is not surprising that, as everywhere in this world, Saudi Arabian patients use antibiotics as a treatment for viral infections. Although the studied hospital is accredited by the Central Board for Accreditation of Healthcare Institutions (CBAHI) and Joint Commission International (JCI), which means that they are implementing standards for infection prevention and control, several additional measures can be put in place to thoroughly solve the AMR problem in Saudi Arabia, which can be summarized in the following points: (I) raising awareness about AMR among the public as well as medical and veterinary personnel. (II) multilevel and countrywide awareness campaigns and the use of social media and webinars addressing all segments and socioeconomic groups in Saudi Arabia to enlighten the people about the dangers of AMR. (III) conducting active and passive educational programs for healthcare workers regarding the importance of hand hygiene compliance to limit the spread of outbreak stains within hospitals, and potentially among the community. (IV) a screening scheme for high-risk patients prior to admission to identify carriers of MDR pathogens and also the isolation of contact precautions. (V) active surveillance; on our part, we tried active surveillance on AMR to track emerging resistance to antibiotics and to identify outbreaks and aid the development of smart treatment guidelines for empirical antibiotic therapy, especially for infections acquired in the community. (VI) up-to-date antibiograms and valid and reliable identification of pathogens through well-equipped microbiology laboratories. (VII) implementation of active antibiotic stewardship guidelines to restrict the irrational use of antibiotics in Saudi Arabia. An updated list of antimicrobials according to their importance in human medicine has been published by WHO and the Advisory Group on Integrated Surveillance of Antimicrobial Resistance (AGSIR) [19].

The hospital where we conducted the study had taken several actions to control the spread of MDR infections. During a colistin-resistant outbreak, for instance, a multidisciplinary committee was constituted. The committee recommended several measures such as environmental screening, training the staff in hand hygiene and the use of precaution protective equipment, disinfecting ICU equipment, restricting ICU visitors, and classifying the patients according to the type of infection and colonization. Several initiatives have been taken to monitor and control the incidence of MDRO infections across the hospital, and in recording the type of organisms and their level of resistance in the state of resistant bacteria. Antimicrobial stewardship programs (ASPs) have been conducted in the hospital to optimize antimicrobial therapy, reduce treatment-related costs, improve clinical outcomes and safety, and reduce or stabilize antimicrobial resistance [20].

Limitations of the study

This study has some limitations. There was some difficulty in retrieving data from the hospital information system. The duration chosen for the retrospective cohort study was not sufficient to indicate correlation, differences, and variance between AMR bacteria and prescription of antibiotics.

## Conclusions

This study showed a high incidence of MDR bacteria in the hospital where the study was conducted. The majority of these were gram-negative. The XDR bacteria found were *A. baumannii*, *K. pneumoniae*, *E. coli, *and *P. aeruginosa*. The most prevalent isolated bacteria included *K. pneumoniae* (18%), *S. aureus* (15.4%), *A. baumannii* (16%), and *P. aeruginosa* (14.2%) in 2016. In 2017, *E. coli* (20.9%), *K. pneumoniae* (17.4%), *S. aureus* (16.5%), and *P. aeruginosa* (13.3%) were the most prevalent. Further research in the field may find ways to reduce the threat of MDR bacterial infections. The hospital will need to implement a more advanced information system that will provide a more comprehensive characterization of factors contributing to the problem.
